# Supplementary material for: Cross-species epigenetic regulation of nucleus accumbens KCNN3 transcripts by excessive ethanol drinking
Source: Transl Psychiatry. 2023 Nov 27;13:364. doi: 10.1038/s41398-023-02676-z (PMC10682415; doi:10.1038/s41398-023-02676-z)
Supplement: Supplementary file 1 — Supplemental methods [file 41398_2023_2676_MOESM1_ESM.docx]

**Supplemental Materials and methods**

**Ethanol self-administration in rhesus macaques**

Male and female rhesus macaques (*n* = 66, *Macaca mulatta*) from seven different cohorts (cohorts 4, 5, 6a, 6b, 7a, 7b, and 10) were included in this study (**Supplemental Table 1**). All of the rhesus macaques were born and reared at the Oregon National Primate Research Center (ONPRC) with their mothers until 2-3 years of age. All subjects were initially selected to minimize relatedness; the average kinship coefficient of all subjects was 0.004. The control subjects were selected by matching age and weight, and then kinship. Controls were housed in the same rooms as the experimental subjects and underwent the same training for awaken blood draws, medical check-ups, and MRI imaging. Controls experienced the same diet, timing and order of experimental phases and had equal experience with the research technicians. However, control monkeys did not have access to ethanol. Instead, controls were yoked to a future ethanol monkey based on weight, and received a quantity of 10% maltose-dextrin solution matched in calories to the previous day's intake of their yoked ethanol monkey. Monkeys were individually housed and ethanol self-administration was induced using schedule-induced polydipsia, as previously described ^1^. For all cohorts, monkeys had open access to 4% ethanol and water (ethanol subjects) or water only (control subjects) for 22 h/day for 12 months (see ^2^ for further details on these seven cohorts). It should be noted that the monkeys in cohort 10 had two cycles of forced abstinence and open access that followed the initial 12 months of open access. Because of the repeated abstinence, samples from monkeys in cohort 10 were limited to *KCNN3* trinucleotide repeat analysis using blood collected prior to ethanol self-administration. While the technicians were not blind to the treatment condition (ethanol or control), the intake data were collected and recorded in an automated fashion by computer and analyzed by individuals who did not interact with or know the drinking status of the monkeys. All of the animal procedures used in this study were approved by the ONPRC IACUC and were performed in accordance with the NIH and the National Resource Council’s *Guide for the Care and Use of Laboratory Animals*.

**Monkey Drinking Phenotypes**

The 49 monkeys with access to ethanol were classified into three different age categories based on their age of first ethanol access and four different drinking categories based on previously described criteria ^3^. There were 15 adolescent (4-5 years of age, ~15-18 human year equivalence; *n* = 6 females), 24 young adult (5-6 years of age, ~18-24 human year equivalence; *n* = 5 females), and 10 mature adult (7-11 years of age, ~25-40 human year equivalence; *n* = 0 females) monkeys. Monkeys were classified as very heavy drinking (VHD) if their average daily ethanol intake was >3 g/kg (~12 drink equivalent in humans) and they consumed >4 g/kg ethanol on ≥ 10% of their open access drinking days. Heavy drinking (HD) monkeys were defined as those that consumed >3 g/kg ethanol on ≥ 20% of their open access drinking days. Binge drinking (BD) monkeys were defined as those that consumed >2 g/kg ethanol on ≥ 55% of their open access drinking days. Low drinking (LD) monkeys were defined as those that did not reach these set thresholds for daily ethanol intake. Classification for cohort 10 was based on their drinking patterns after 12 months of open access. We have previously confirmed that the ethanol drinking behaviors did not reflect general differences in thirst ^4^.

**Ethanol dependence and two-bottle choice drinking in C57BL/6J mice**

Sixty adult male C57BL/6J mice were purchased from Jackson Laboratory (Bar Harbor, ME) at ~7 weeks of age. Mice were individually housed in a temperature and humidity controlled environment and kept on a 12 h light/dark cycle. Food and water were available *ad libitum* during all procedures. The Medical University of South Carolina Institutional Animal Care and Use Committee approved all procedures in accordance with NIH guidelines for the humane care and use of laboratory animals. Mice were randomly assigned to the experimental groups. To establish baseline drinking, half of the mice consumed ethanol in their home cage using a 2-bottle choice (15% ethanol (v/v) vs. water) long-access (22 h) protocol for five weeks. Control mice consumed water in their home cage during this phase. Half of the control and ethanol drinking mice then underwent 4 repeated weekly cycles of chronic intermittent ethanol (CIE) exposure in vapor inhalation chambers, alternated with weekly home cage drinking sessions [2 (water vs ethanol drinking) x 2 (air vs ethanol vapor inhalation) experimental design; *n* = 15 mice/group] with 72 h in between CIE exposure and access to ethanol drinking bottles in their home cages. Ethanol vapor exposure was delivered in Plexiglas inhalation chambers as previously described ^5^. CIE treatment consists of sixteen hours of vapor exposure followed by eight h of withdrawal. Chamber ethanol concentrations were monitored daily and air flow was adjusted to maintain ethanol concentrations within a range that yields stable blood ethanol levels (175–225 mg/dl) throughout exposure. Prior to entry into the ethanol chambers, EtOH mice were administered ethanol (1.6 g/kg; 8% w/v; i.p.; 20 ml/kg dose volume) and the alcohol dehydrogenase inhibitor pyrazole (1 mmol/kg). Control mice were handled similarly, but received injections of saline and pyrazole. Seventy-two h following the last vapor chamber exposure, mice were given limited access to ethanol or water for 2-3 days prior to sacrifice and tissue collection.

**Genomic DNA and total RNA isolation**

After the 12 month open access period, a detailed necropsy protocol was used to systematically collect tissues from all macaques ^6^. Monkeys were sedated with ketamine (15 mg/kg), and then the animals were brought into a surgical plane of anesthesia with intravenous administration of sodium pentobarbital (30–50 mg/kg, i.v.). Following extraction, the entire brain was blocked or sectioned into slices that were fresh frozen and stored at -80^o^ C. The NAcC was later dissected from frozen brain slices. The typical postmortem interval for brain extractions was < 5 minutes. Mouse brains were rapidly removed and placed in ice-cold saline before blocking 1-mm thick sections using a mouse brain block (ASI Instruments, Inc., Warren, MI, USA). A 1-mm tissue punch (Ted Pella, Inc., Redding, CA, USA) was used to extract the NAcC. Genomic DNA and RNA were extracted from male and female monkey and male mouse NAcC samples using the All Prep DNA/RNA/miRNA Universal kit (QIAGEN Sciences Inc, Germantown, MD) following the manufacturer’s recommendations. Blood samples drawn from macaques prior to ethanol self-administration were used for CAG repeat analysis. Briefly, blood was collected in EDTA tubes and DNA was isolated using QIAamp DNA mini kit following manufacturer’s instructions (QIAGEN Sciences Inc).

**Trinucleotide repeat analysis**

Blood DNA was used to analyze the number of CAG repeats within the second *KCNN3* CAG repeat region as previously described ^7^. The primers (Forward: CAGCAGCCCCTGGGACCCTCG, Reverse: GGAGTTGGGCGAGCTGAGACAG) generated amplicons ranging between 112 bp and 178 bp, depending on the number of CAG repeats present. The PCR products were applied to an ABI3730 XL DNA Analyzer for separation and detection, incorporating a 600LIZ size standard with the PCR products at a 1:1 ratio (Applied Biosystems Inc.). The output files were visualized and product sizes were determined using Gene Mapper 4.0 software (Applied Biosystems Inc.). The sequence content of the amplification products was confirmed by PCR amplifying DNA from individuals with homozygous genotypes, and by direct DNA sequencing of PCR products. We found that the first exon 1 CAG repeat was not variable in rhesus macaques; thus, these studies focused on the second exon 1 CAG repeat length.

**Bisulfite amplicon sequencing**

Bisulfite amplicon sequencing was used to measure the DNAm rates of a DMR within the *KCNN3* promoter region using NAcC tissue from macaques and mice. Correction for bisulfite-converted PCR bias was carried out as described by Moskalev et al. ^8^. Methylated and unmethylated human gDNAs (Zymo Research, Irvine, CA) were bisulfite-converted using the EZ DNA Methylation-Gold kit (Zymo Research, Irvine, CA), according to the manufacturer’s instructions. The bisulfite converted DNAs were combined to create a series of methylation rate standards ranging from 100% to 0% methylated. gDNA (500ng) from rhesus and mouse NAcC was bisulfite converted following the same protocol. Primers were designed to amplify a 646bp region of the *KCNN3* within exon 1A and intron 1 in human, rhesus macaque and mice. Because of the length of the region, two sets of primers were designed to cover the whole region (**Supplemental Table 2**). Amplification was carried out in the C1000 Thermal Cycler (Bio-Rad, Hercules, CA) using 20 ng of bisulfite-treated DNA per PCR reaction. Amplification was carried out as follows: Phase 1: 10 cycles of 94^o^ C for 30sec., 68^o^ C for 1min., with a touchdown decrease of 1^o^C per cycle. Phase 2: 28 cycles of 94^o^C for 30sec. and 58^o^C for 45sec. Libraries were prepared using the NETflex DNA Sequencing Kit (BIOO Scientific, Austin, TX) according to manufacturer’s instructions. The libraries were evaluated using an Agilent 2100 Bioanalyzer (Agilent Technologies, Palo Alto, CA) and were normalized to 2nM with 10 mM Tris-HCl. The libraries were then pooled and sequenced on a MiSeq (Illumina, Inc. San Diego, CA) by the Molecular & Cellular Biology Core, (ONPRC, Beaverton, OR) to generate 250-base paired-end reads. The reads were trimmed using Trim Galore and aligned to the corresponding reference genome (Rhesus: MacaM ^9^ and Mouse: GRCm38.p3; https://www.ncbi.nlm.nih.gov/assembly/GCF_000001635.20/) using Bismark ^10^. M-bias plots were then generated ^11^, and reads were trimmed further as needed. Bismark alignment data was converted to CpG methylation rate using the Bismark methylation extractor ^10^ and custom scripts.

To assess the linearity of the PCR amplification we plotted the observed versus expected methylation rates obtained from the methylated DNA standard dilution series ^8^. Next, a hyperbolic function was applied to correct for PCR-bias obtaining a good linear fit (r_Pair-1_^2^=0.94; r_Pair-2_^2^=0.94; r_Pair-3_^2^=0.98; **Supplemental Fig. 5**). Code will be made accessible upon request by investigators.

**RNA isolation and reverse transcription**

The NAcC RNA quantity and quality was evaluated on a 2100 Bioanalyzer (Agilent Technologies, Santa Clara, CA). The Fluidigm Reverse Transcription Master Mix (Fluidigm, Inc., San Francisco, CA) was used to reverse-transcribe 100 ng of each RNA sample following the manufacturer’s instructions. Briefly, 1 μl of 100 ng of RNA was combined with 1 μl of Reverse Transcription Master Mix and 3 μl of RNase-free water. The reactions were incubated at 25 C for 2 min, followed by 42 C for 30 min and 85 C for 5 min. Next, 1.25 μl of cDNA was pre-amplified using 1 μl of PreAmp master mix, 0.5 μl of pooled primers (at 500 nM) and 2.25 μl of RNase-free water. The Pre-Amplification conditions were 95 C for 2 min, followed by 10 cycles at 95 C for 15 s and 60 C for 4 min. To remove unincorporated primers, the reaction were mixed with 0.2 μl Exonuclease I reaction buffer, 0.4 μl Exonuclease I (20 Units/ml) and 1.4 μl of RNase-free water. The reactions were incubated at 37 C for 30 min followed by 80 C for 15 min. The reactions were diluted (10x) with 43 μl of TE buffer (TEKnova, Hollister, CA).

**High-throughput real time PCR**

qPCR was performed using the BioMark™ HD System and the 96.96 GE Dynamic Arrays (Fluidigm, Inc., San Francisco, CA) in triplicates assays. 5 μL of Fluidigm sample premix consisted of 2.25 μL of 10x diluted pre-amplified cDNA, 0.25 μL of 20x SG loading reagent (Fluidigm), 2.5 μL of Sso Fast Eva Green Mastermix (Bio-Rad). Each 5 μL assay premix consisted of 0.25 μL of 100 μM primers (final concentration 500 nM primers), 2.5 μL 2x Assay loading reagent (Fluidigm) and 2.25 μL of 1x DNA suspension buffer (TEKnova, Hollister, CA). The samples and assays were mixed using the Nanoflex IFC controller (Fluidigm). Thermal qPCR conditions were: 95°C for 60 s, 35 cycles of 95°C for 5 s, and 60°C for 20 s plus melting curve analysis. Data was processed by automatic threshold for each assay, with derivative baseline correction using BioMark Real-Time PCR Analysis Software 3.1.2 (Fluidigm). The quality threshold was set at the default of 0.65.

The primer sequences are described in **Supplemental Table 3**. Since most of the alternative transcript variants are not annotated in the Rhesus or mouse genome, we used the human annotations to design the primers, then identified the homologous sequence in the rhesus macaque (MacaM) ^9^ and mouse (GRCm38.p3) genome. The mRNA expression levels were normalized using the phosphoglycerate kinase *(PGK1)* gene. This gene was demonstrated to be a reliable control for brain gene expression ^12^. We also previously confirmed that different levels of ethanol use did not affect its expression ^13^. The monkeys 10171 (male) and 10068 (female) and mouse 16C control subjects were used as a reference sample for comparison.

**Western blot analysis**

After extraction, tissue samples containing the NAcC extracted from female control and long-term drinking rhesus macaques (*n* = 5/group) were snap frozen, shipped overnight on dry ice, and homogenized in 100 µl of ice-cold homogenization buffer (50 mM Tris-HCl, 50 mM NaCl, 10 mM EGTA, 5 mM EDTA; 2 mM sodium pyrophosphate, 1 mM activated sodium orthovanadate, 0.2 mM AEBSF, 1 µg/ml aprotinin, 1 mM benzamide, 10 µg/ml leupeptin, 10 µg/ml pepstatin, pH 7.5). Samples were probe sonicated for ~ 5 sec and centrifuged at 23,100 × g for 30 min at 4°C. The resulting supernatant was removed and the pellet was resuspended in 2% lauryl dodecyl sulfate (LDS) and probe sonicated for ~ 5 sec. An aliquot was taken for determination of protein concentration by the bicinchoninic acid assay (Pierce Biotechnology, Inc., Rockford, IL). Samples were diluted with NuPAGE 4X LDS sample loading buffer (Invitrogen Corp., Carlsbad, CA; pH 8.5) containing 50 mM dithiothreitol, and samples were denatured for 10 min at 70°C. We first performed a series of western blots using different titrations of sample and antibody to establish the linear range for K_Ca_2.3 (Alomone Labs, Jerusalem, Israel; Catalog #: APC-025; epitope AA 2-21 of human K_Ca_2.3) in primate tissue samples. Specificity of this antibody for K_Ca_2.3 channels has been confirmed in conditional K_Ca_2.3 knockout mice ^14^. Ten µg of each experimental sample was separated using the Bis-Tris (375 mM resolving buffer and 125 mM stacking buffer, pH 6.4; 7.5% acrylamide) discontinuous buffer system with MOPS electrophoresis buffer (50 mM MOPS, 50 mM Tris, 0.1% SDS, 1 mM EDTA, pH 7.7). Protein was then transferred to Immobilon-P PVDF membranes (Millipore, Bedford, MA) using a semi-dry transfer apparatus (Bio-Rad Laboratories, Hercules, CA). Blots were then washed with phosphate-buffered saline containing 0.1% Tween 20 (PBST) and blocked with PBST containing 5% nonfat dried milk (NFDM) for 1 h at room temperature with agitation. The membranes were then incubated overnight at 4°C with primary antibody diluted 1:4000 in PBST containing 0.5% NFDM and washed in PBST prior to 1 h incubation at room temperature with horseradish peroxidase conjugated secondary antibody diluted 1:2000 in PBST. Membranes received a final wash in PBST and the antigen-antibody complex was detected by enhanced chemiluminescence using a ChemiDoc MP Imaging system (Bio-Rad Laboratories, Hercules, CA). The bands in the experimental samples were background subtracted and quantified by mean optical density using computer-assisted densitometry with Image Lab software (v4.0.1, Bio-Rad Laboratories) with the experimenter blind to the ethanol drinking groups. Because controls (e.g., actin, GAPDH) used to normalize protein loading in western blot experiments can cause quantitation errors ^15-17^, we used normalized to a total protein following our published methods in monkey tissue ^18^.

**Statistical Analysis**

Data from heavy and very heavy drinking monkeys were combined due to small sample sizes in the transcript analysis and bisulfite sequencing studies. All statistical analyses were carried out using IBM SPSS Statistics (Armonk, NY) except where noted, with values α<0.05. The Shapiro-Wilk test (appropriate for small sample sizes) was used to assess the normality of the average methylation rate, mRNA expression rate, and K_Ca_2.3 protein expression level per comparison group. All variables analyzed followed a normal distribution. Welch’s one-way ANOVA was used to compare the difference in average methylation between controls and ethanol drinkers with Games-Howell post-hoc tests. One-way ANOVA was used to compare mRNA relative expression levels between groups. Prior to applying one-way ANOVA, Levene's test was used to test homogeneous variance assumption for parametric methods. When heterogeneous variance was detected, we used the nonparametric Kruskal-Wallis test. Bonferroni or Tukey correction for the multiple comparisons were used to correct the overall type I error rate. Two-tailed independent t-test was used to compare the difference in average methylation rate between controls and dependent mice. Based on the Levene’s test for homogeneous variance, we used the appropriate p-value (homogeneous or heterogeneous variance). The allele frequency distribution of CAG trinucleotide repeats between controls and drinking monkeys was compared using the Kruskal-Wallis test (GraphPad Prism software, version 7.04, La Jolla, CA). Normalized western blot data were analyzed by a two-tailed t-test in Prism. Ethanol drinking data in mice were analyzed by a repeated-measures mixed linear model with a Tukey post-hoc test (SAS Institute, Cary, NC, USA).

**SUPPLEMENTAL REFERENCES**

1. Grant KA, Leng X, Green HL, Szeliga KT, Rogers LS, Gonzales SW. Drinking typography established by scheduled induction predicts chronic heavy drinking in a monkey model of ethanol self-administration. *Alcohol Clin Exp Res* 2008; **32**(10)**:** 1824-1838.

2. Baker EJ, Walter NA, Salo A, Rivas Perea P, Moore S, Gonzales S *et al.* Identifying Future Drinkers: Behavioral Analysis of Monkeys Initiating Drinking to Intoxication is Predictive of Future Drinking Classification. *Alcohol Clin Exp Res* 2017; **41**(3)**:** 626-636.

3. Baker EJ, Farro J, Gonzales S, Helms C, Grant KA. Chronic alcohol self-administration in monkeys shows long-term quantity/frequency categorical stability. *Alcohol Clin Exp Res* 2014; **38**(11)**:** 2835-2843.

4. Cervera-Juanes R, Wilhem LJ, Park B, Lee R, Locke J, Helms C *et al.* MAOA expression predicts vulnerability for alcohol use. *Mol Psychiatry* 2016; **21**(4)**:** 472-479.

5. Padula AE, Griffin WC, 3rd, Lopez MF, Nimitvilai S, Cannady R, McGuier NS *et al.* KCNN Genes that Encode Small-Conductance Ca2+-Activated K+ Channels Influence Alcohol and Drug Addiction. *Neuropsychopharmacology* 2015; **40**(8)**:** 1928-1939.

6. Davenport AT, Grant KA, Szeliga KT, Friedman DP, Daunais JB. Standardized method for the harvest of nonhuman primate tissue optimized for multiple modes of analyses. *Cell Tissue Bank* 2014; **15**(1)**:** 99-110.

7. Curtain R, Sundholm J, Lea R, Ovcaric M, MacMillan J, Griffiths L. Association analysis of a highly polymorphic CAG Repeat in the human potassium channel gene KCNN3 and migraine susceptibility. *BMC medical genetics* 2005; **6:** 32.

8. Moskalev EA, Zavgorodnij MG, Majorova SP, Vorobjev IA, Jandaghi P, Bure IV *et al.* Correction of PCR-bias in quantitative DNA methylation studies by means of cubic polynomial regression. *Nucleic acids research* 2011; **39**(11)**:** e77.

9. Zimin AV, Cornish AS, Maudhoo MD, Gibbs RM, Zhang X, Pandey S *et al.* A new rhesus macaque assembly and annotation for next-generation sequencing analyses. *Biol Direct* 2014; **9**(1)**:** 20.

10. Krueger F, Andrews SR. Bismark: a flexible aligner and methylation caller for Bisulfite-Seq applications. *Bioinformatics* 2011; **27**(11)**:** 1571-1572.

11. Hansen KD, Langmead B, Irizarry RA. BSmooth: from whole genome bisulfite sequencing reads to differentially methylated regions. *Genome Biol* 2012; **13**(10)**:** R83.

12. Boda E, Pini A, Hoxha E, Parolisi R, Tempia F. Selection of reference genes for quantitative real-time RT-PCR studies in mouse brain. *J Mol Neurosci* 2009; **37**(3)**:** 238-253.

13. Cervera-Juanes R, Wilhelm LJ, Park B, Grant KA, Ferguson B. Alcohol-dose-dependent DNA methylation and expression in the nucleus accumbens identifies coordinated regulation of synaptic genes. *Translational psychiatry* 2017; **7**(1)**:** e994.

14. Bond CT, Herson PS, Strassmaier T, Hammond R, Stackman R, Maylie J *et al.* Small conductance Ca2+-activated K+ channel knock-out mice reveal the identity of calcium-dependent afterhyperpolarization currents. *J Neurosci* 2004; **24**(23)**:** 5301-5306.

15. Aldridge GM, Podrebarac DM, Greenough WT, Weiler IJ. The use of total protein stains as loading controls: an alternative to high-abundance single-protein controls in semi-quantitative immunoblotting. *J Neurosci Methods* 2008; **172**(2)**:** 250-254.

16. Dittmer A, Dittmer J. Beta-actin is not a reliable loading control in Western blot analysis. *Electrophoresis* 2006; **27**(14)**:** 2844-2845.

17. Welinder C, Ekblad L. Coomassie staining as loading control in Western blot analysis. *Journal of proteome research* 2011; **10**(3)**:** 1416-1419.

18. Nimitvilai S, Uys JD, Woodward JJ, Randall PK, Ball LE, Williams RW *et al.* Orbitofrontal Neuroadaptations and Cross-Species Synaptic Biomarkers in Heavy-Drinking Macaques. *J Neurosci* 2017; **37**(13)**:** 3646-3660.
