# Supplementary material for: Cross-species epigenetic regulation of nucleus accumbens KCNN3 transcripts by excessive ethanol drinking
Source: Transl Psychiatry. 2023 Nov 27;13:364. doi: 10.1038/s41398-023-02676-z (PMC10682415; doi:10.1038/s41398-023-02676-z)
Supplement: Supplementary file 2 — Supplemental table 1 [file 41398_2023_2676_MOESM2_ESM.docx]

**Supplemental Table 1.** Descriptions of the male and female rhesus macaques used in these studies. Adol, adolescent; BD, binge drinking; BS, bisulfite sequencing analysis; CAG, trinucleotide repeat genotyping analysis; DOB, date of birth; HD, heavy drinking; LD, low drinking; MA, mature adult; TV, *KCNN3* transcript variant analysis; VHD, very heavy drinking; WB, K_Ca_2.3 channel western blotting analysis; YA, young adult.

| **MATRR ID** | **Cohort** | **Sex** | **DOB** | **EtOH Intake (g/kg/22 h)** | **Drinking Category** | **Age (in years) at start of EtOH Induction** | **Age**  **Category** | **Study Inclusion** | | | |
| --- | --- | --- | --- | --- | --- | --- | --- | --- | --- | --- | --- |
|  |  |  |  |  |  |  |  | **CAG** | **TV** | **BS** | **WB** |
| 10048 | 4 | M | 07/27/1998 | 1.25 | LD | 9.85 | MA | Yes |  | Yes |  |
| 10049 | 4 | M | 08/11/1998 | 2.62 | HD | 9.81 | MA | Yes | Yes | Yes |  |
| 10051 | 4 | M | 03/15/1999 | 2.65 | BD | 9.22 | MA | Yes |  |  |  |
| 10052 | 4 | M | 04/01/1999 | 0.47 | LD | 9.18 | MA | Yes | Yes | Yes |  |
| 10054 | 4 | M | 05/24/2000 | 2.51 | BD | 8.03 | MA | Yes |  |  |  |
| 10055 | 4 | M | 01/01/2000 | 1.03 | LD | 8.43 | MA | Yes | Yes | Yes |  |
| 10056 | 4 | M | 06/01/2000 | 1.56 | LD | 8.01 | MA | Yes | Yes | Yes |  |
| 10057 | 4 | M | 05/15/2001 | 2.29 | BD | 7.05 | MA | Yes |  |  |  |
| 10058 | 4 | M | 04/01/2001 | 1.99 | LD | 7.18 | MA | Yes | Yes |  |  |
| 10059 | 4 | M | 04/01/2001 | 2.43 | BD | 7.18 | MA | Yes |  |  |  |
| 10061 | 5 | M | 02/01/2003 | 3.78 | VHD | 5.85 | YA | Yes | Yes | Yes |  |
| 10062 | 5 | M | 01/01/2003 | 4.09 | VHD | 5.94 | YA |  |  | Yes |  |
| 10063 | 5 | M | 04/01/2003 | 3.09 | VHD | 5.69 | YA |  |  | Yes |  |
| 10064 | 5 | M | 04/15/2003 | 2.74 | HD | 5.65 | YA | Yes |  | Yes |  |
| 10065 | 5 | M | 01/01/2003 | 3.09 | HD | 5.94 | YA | Yes | Yes | Yes |  |
| 10066 | 5 | M | 03/01/2003 | 3.27 | VHD | 5.77 | YA | Yes | Yes | Yes |  |
| 10067 | 5 | M | 02/15/2003 | 3.08 | HD | 5.81 | YA | Yes |  | Yes |  |
| 10167 | 5 | M | 02/14/2002 | - | CTRL | - | - |  |  | Yes |  |
| 10169 | 5 | M | 03/11/2004 | - | CTRL | - | - |  |  | Yes |  |
| 10170 | 5 | M | 06/11/2004 | - | CTRL | - | - |  | Yes | Yes |  |
| 10171 | 5 | M | 10/30/2004 | - | CTRL | - | - |  | Yes | Yes |  |
| 10208 | 10 | M | 06/14/2007 | 2.29 | BD | 5.88 | YA | Yes |  |  |  |
| 10209 | 10 | M | 06/28/2007 | 2.39 | BD | 5.84 | YA | Yes |  |  |  |
| 10210 | 10 | M | 03/01/2007 | 1.55 | LD | 6.16 | YA | Yes |  |  |  |
| 10211 | 10 | M | 04/22/2008 | 2.11 | BD | 5.02 | YA | Yes |  |  |  |
| 10212 | 10 | M | 04/15/2008 | 2.32 | HD | 5.04 | YA | Yes |  |  |  |
| 10213 | 10 | M | 08/01/2008 | 1.29 | LD | 4.74 | Adol | Yes |  |  |  |
| 10214 | 10 | M | 04/15/2008 | 4.22 | VHD | 5.04 | YA | Yes |  |  |  |
| 10215 | 10 | M | 04/01/2008 | 3.19 | VHD | 5.08 | YA | Yes |  |  |  |
| 10068 | 6a | F | 05/01/2006 | n/a | CTRL | - | - |  |  | Yes | Yes |
| 10069 | 6a | F | 06/08/2006 | 5.15 | VHD | 4.14 | Adol | Yes | Yes |  |  |
| 10070 | 6a | F | 06/15/2006 | 3.92 | VHD | 4.12 | Adol | Yes | Yes | Yes |  |
| 10071 | 6a | F | 04/15/2006 | - | CTRL | - | - |  | Yes | Yes | Yes |
| 10076 | 6a | F | 05/01/2006 | - | CTRL | - | - |  | Yes | Yes | Yes |
| 10078 | 6a | F | 09/26/2006 | 5.04 | VHD | 3.84 | Adol | Yes | Yes | Yes | Yes |
| 10079 | 6a | F | 06/01/2006 | 4.02 | VHD | 4.16 | Adol | Yes | Yes | Yes | Yes |
| 10080 | 6a | F | 06/01/2006 | 3.27 | VHD | 4.16 | Adol | Yes | Yes | Yes | Yes |
| 10081 | 6a | F | 06/01/2006 | 4 | VHD | 4.16 | Adol | Yes | Yes |  | Yes |
| 10072 | 6b | F | 04/01/2006 | 1.05 | LD | 5.83 | YA | Yes |  |  | Yes |
| 10073 | 6b | F | 06/15/2006 | 3.94 | VHD | 5.63 | YA | Yes | Yes | Yes |  |
| 10074 | 6b | F | 07/01/2006 | 1.71 | LD | 5.58 | YA | Yes |  |  |  |
| 10075 | 6b | F | 04/01/2006 | 2.78 | HD | 5.83 | YA | Yes |  |  |  |
| 10077 | 6b | F | 01/15/2006 | 1.34 | LD | 6.04 | YA | Yes |  |  |  |
| 10186 | 6b | F | 05/01/2006 | - | CTRL | - | - |  | Yes | Yes | Yes |
| 10187 | 6b | F | 03/15/2006 | - | CTRL | - | - |  | Yes |  | Yes |
| 10188 | 6b | F | 08/11/2006 | - | CTRL | - | - |  | Yes |  |  |
| 10087 | 7a | M | 04/23/2006 | 2.36 | BD | 4.36 | Adol | Yes |  |  |  |
| 10088 | 7a | M | 03/06/2006 | 2.89 | HD | 4.49 | Adol | Yes | Yes |  |  |
| 10089 | 7a | M | 01/06/2006 | 2 | LD | 4.65 | Adol | Yes | Yes | Yes |  |
| 10090 | 7a | M | 05/30/2006 | 1.85 | LD | 4.25 | Adol | Yes | Yes | Yes |  |
| 10091 | 7a | M | 04/27/2006 | 3.15 | VHD | 4.34 | Adol | Yes | Yes |  |  |
| 10092 | 7a | M | 05/15/2006 | 1.9 | LD | 4.29 | Adol | Yes | Yes | Yes |  |
| 10093 | 7a | M | 03/15/2006 | - | CTRL | - | - |  | Yes | Yes |  |
| 10094 | 7a | M | 03/15/2006 | - | CTRL | - | - |  | Yes | Yes |  |
| 10096 | 7a | M | 04/15/2006 | - | CTRL | - | - |  | Yes | Yes |  |
| 10097 | 7a | M | 05/01/2006 | 3.03 | HD | 4.33 | Adol | Yes | Yes | Yes |  |
| 10098 | 7a | M | 09/05/2006 | 3.32 | VHD | 3.99 | Adol | Yes | Yes |  |  |
| 10082 | 7b | M | 12/01/2004 | 2.42 | HD | 6.30 | YA | Yes | Yes | Yes |  |
| 10083 | 7b | M | 06/01/2005 | 1.44 | LD | 5.80 | YA | Yes | Yes | Yes |  |
| 10084 | 7b | M | 07/21/2005 | 1.85 | LD | 5.66 | YA | Yes | Yes | Yes |  |
| 10085 | 7b | M | 05/06/2005 | 2.07 | LD | 5.87 | YA | Yes | Yes |  |  |
| 10086 | 7b | M | 07/26/2005 | 2.28 | BD | 5.64 | YA | Yes |  |  |  |
| 10182 | 7b | M | 03/13/2005 | - | CTRL | - | - |  | Yes | Yes |  |
| 10183 | 7b | M | 06/01/2005 | - | CTRL | - | - |  | Yes | Yes |  |
| 10184 | 7b | M | 04/01/2005 | - | CTRL | - | - |  | Yes |  |  |
| 10185 | 7b | M | 02/01/2005 | - | CTRL | - | - |  | Yes |  |  |
