# Supplementary material for: Cross-species epigenetic regulation of nucleus accumbens KCNN3 transcripts by excessive ethanol drinking
Source: Transl Psychiatry. 2023 Nov 27;13:364. doi: 10.1038/s41398-023-02676-z (PMC10682415; doi:10.1038/s41398-023-02676-z)
Supplement: Supplementary file 3 — Supplemental table 2 [file 41398_2023_2676_MOESM3_ESM.docx]

**Supplemental Table 2**. Primers used for bisulfite amplicon sequencing.

| **Species** | **Genomic sequence** | **Primer** | **Position*** | **Sequence** | **Amplicon length** |
| --- | --- | --- | --- | --- | --- |
| Macaca mulatta | NC_027893.1 | Pair 1-Forward | 129559145 | TTTTTGAGTATAAATTTTAAGAGAGTT | 261 bp |
|  |  | Pair 1-Reverse | 129559405 | AAAACAACCATACCCACCAAAC |  |
|  |  | Pair 2-Forward | 129559386 | TTGGTGGGTATGGTTGTTTTT | 275 bp |
|  |  | Pair 2-Reverse | 129559660 | TCTCCAAATTCCACAACTACAAATA |  |
|  |  | Pair 3-Forward | 129559511 | GGTTGAGGGGTTTTATGATTTTAT | 280 bp |
|  |  | Pair 3-Reverse | 129559790 | CACCCTAACCTACTACACTCCTCTC |  |
| Mus musculus | NC_000069.6 | Pair 1-Forward | 726 | TTGGTGGGTATGGTTATTTT | 275 bp |
|  |  | Pair 1-Reverse | 1000 | CTCCAAATTCCACAACCACAAAT |  |
|  |  | Pair 2-Forward | 981 | TTTTTGAGTATAAATTTTAAGATAGTT | 261 bp |
|  |  | Pair 2-Reverse | 1241 | AAAATAACCATACCCACCAAAC |  |

*Position from the transcription start site (TSS).
