# Supplementary material for: Cross-species epigenetic regulation of nucleus accumbens KCNN3 transcripts by excessive ethanol drinking
Source: Transl Psychiatry. 2023 Nov 27;13:364. doi: 10.1038/s41398-023-02676-z (PMC10682415; doi:10.1038/s41398-023-02676-z)
Supplement: Supplementary file 4 — Supplemental table 3 [file 41398_2023_2676_MOESM4_ESM.docx]

**Supplemental Table 3**. Nucleotide sequences of primers used for quantitative PCR.

| **Primer** | **MacaM Genomic Position** | **mm10 Genomic Position** | **Human transcript (GRCh38)** | **Sequence (MacaM)** | **Sequence (mm10)^** | **Amplicon length** |
| --- | --- | --- | --- | --- | --- | --- |
| SK3_ex7/8-Forward | 128909919 | 89662751 | NM_002249.5 (exons 7-8); NM_001204087.1; AY138900.1 | GACCAAGCCAACACCCTGG | GACCAAGCCAACACCCTGG | 104 bp |
| SK3_ex7/8-Reverse | 128909815 | 89667136 |  | CCAATCTGCTTCTCCAGGTC | CCAATCTGCTTtTCtAGaTC |  |
| SK3_ex1B-Forward | 129129358 | 89013104 | AY138900.1  exons 1-2 | ACTGCTCCAGCCTCTCAGTC | ctTGCTCCAGCCcTgCTCAGcC | 158 bp |
| SK3_ex1B-Reverse | 129083314 | 89564941 |  | TGTGGTAGGCGATGATCAAG | TGTGaTAGGCGATGATCAAa |  |
| SK3_ex4-Forward | 129032187 | 89609619 | NM_001204087.1 exons 3-4 | ACTTCAACACCCGCTTTGTC | ACTTCAACACCCGATTCGTC | 137 bp |
| SK3_ex4-Reverse | Not annotated* | NA |  | CTGGGCTGGTGATTCAGGAC | N/A^#^ |  |

*Although the homologous exon 4 of the human TV NM_001204087.1 is not annotated in MacaM, it is in the most updated version of the macaque genome (Mmul8).

^lower case letters represent non-conserved nucleotides between macaques and mice.

^#^sequence not annotated in the mouse genome, we used the macaque primer instead.
