# Supplementary material for: Cross-species epigenetic regulation of nucleus accumbens KCNN3 transcripts by excessive ethanol drinking
Source: Transl Psychiatry. 2023 Nov 27;13:364. doi: 10.1038/s41398-023-02676-z (PMC10682415; doi:10.1038/s41398-023-02676-z)
Supplement: Supplementary file 5 — Supplemental table 4 [file 41398_2023_2676_MOESM5_ESM.docx]

**Supplemental Table 4**. *KCNN3* transcript variants, protein isoforms, and channel function.

| **Transcript Variant** | **Unique exons** | **Protein isoform** | **Changes in protein structure** | **Altered Channel Function** |
| --- | --- | --- | --- | --- |
| *SK3_1A* (NM_002249.5) | 1A | 1: Full-Length (NP_002240.3) | None | None |
| *SK3_1B* (NM_170782.2) | 1B | 3 (NP_740752.1) | Lacks S1 transmembrane domain; Missing aa1-318 | Dominant-negative |
| *SK3_1C* (AY138900.1) | 1C | 2 (AAN46636.1) | Lacks S1 transmembrane domain; missing aa1-310; 311-315: WGLYS → MERPI | Dominant-negative; Very low CNS expression |
| *SK3_ex4* (NM_001204087.1) | 4 | SK3_ex4 (NP_001191016) | 15 amino-acid insert in the pore-forming loop | Apamin-insensitive |
