# Supplementary material for: Cross-species epigenetic regulation of nucleus accumbens KCNN3 transcripts by excessive ethanol drinking
Source: Transl Psychiatry. 2023 Nov 27;13:364. doi: 10.1038/s41398-023-02676-z (PMC10682415; doi:10.1038/s41398-023-02676-z)
Supplement: Supplementary file 6 — Supplemental figures [file 41398_2023_2676_MOESM6_ESM.pdf]

Supplemental Figure 1

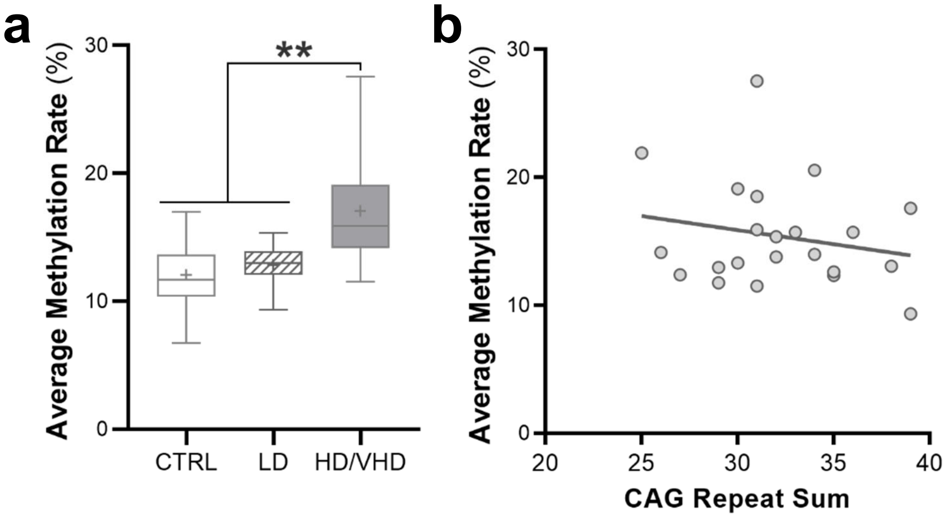

Supplemental Fig. 1. Averaged methylation rate (%) grouped by drinking class for all CpGs in male and female rhesus macaques. **a** The methylation rate was significantly higher in the HD/VHD (N = 15) compared to control (N = 13) and LD (N = 9) macaques ($F(2, 34) = 9.860$, $p = 0.0004$, Tukey post-hoc, $^{**}p < 0.01$). **b** Lack of correlation between average methylation rate and CAG repeat sum ($p = 0.3563$)

Supplemental Figure 2

```
                                                              Hs  154,869,051  CCTTTGAGTAC  154,869,041
                                                              Rm  129,130,369  CCTTTGAGTAC  129,130,359
                                                              Mm   89,521,405  CCTTTGAGTAC   89,521,395

Hs 154,869,042  AAACCCCAAGAGAGCTCGGTCTCTATCACCATAACAACAATTCCAAACATCCCAAAAATC  154,869,101
Rm 129,130,360  AAACCCCAAGAGAGCTCGGTCTCTATCACCATAACAACAATTCCAAACATCCCAAAAATC  129,130,419
Mm  89,521,394  AAACCCCAAGACAGTTCGGTCTCTATCACCATAACAACAATTCCAAACATCCCAAAAATC   89,521,335

Hs 154,869,102  AGAGCATAGTCACTCAGTCGCTTTCTCTTTTCAAACAGGGCCCTCCTGTGTCCCAGCTTA  154,869,161
Rm 129,130,420  AGAGCATAGTCACTCAGTCGCTTTCTCTTTTCAAACAGGGCCCTCCTGTGTCCCAGCTTA  129,130,479
Mm  89,521,334  AGAGCATAGTCACTCAGTCGCTTTCTCTTTTCAAACAGGGCCCTCCTGTGTCCCAGCTTA   89,521,275

Hs 154,869,162  TAGCCAATGTTTTGGTTTTTCCGCTTGTTGGCTTTGGGGAAGGTGGTGCTGCTGGCGGTG  154,869,221
Rm 129,130,480  TAGCCAATGTTTTGGTTTTTCCGCTTGTTGGCTTTGGGGAAGGTGGTGCTGCTGGCGGTG  129,130,539
Mm  89,521,274  TAGCCAATGTTTTGGTTTTTCCGCTTGTTGGCTTTGGGGAAGGTGGTGCTGCCGGCCGTG   89,521,215

Hs 154,869,222  GTGCCGGCATGCTGGTGGTTGTGGGTGGCATTAGGGTGATGGAGCAGGGTCTGGTGGGCA  154,869,281
Rm 129,130,541  GTGCCGGCATGCTGGTGGTTGTGGGTGGCATTAGGGTGATGGAGCAGGGTCTGGTGGGCA  129,130,599
Mm  89,521,214  GTGCCGGCATGCTGGTGGTTGTGGGTAGCGTTGGGGTGATGGAGCAGAGTCTGGTGGGCA   89,521,155

Hs 154,869,282  TGGTTGTCCTCCCGGGAGGAGATGACGATCTCCGGGGGGTTGCTAGGGCTGAAAAGCTGG  154,869,341
Rm 129,130,600  TGGTTGTCCTCCCGGGAGGAGATGACGATCTCCGGGGGGTTGCTAGGACTGAAAAGCTGG  129,130,659
Mm  89,521,154  TGGTTATCCTCCCTGGAGGAGATGATAATCTCTGGGGGGTTGCTGGGACTGAAGAGCTGG   89,521,095

Hs 154,869,342  AGGGGTTGGCCCTCAGTCTCGGCCTCGATGAGGTTCCTCCGGGAGGCGCTGAGGCGGCTG  154,869,401
Rm 129,130,660  AGGGGTTGGCCCTCAGTCTCCGCCTCGATGAGGTTCCTCCGGGAGGCGCTGAGGCGGCTG  129,130,719
Mm  89,521,094  AGGGGTTGGCCCTCAGGCTCAGCCTCGATAAGGTTTCTCCGAGAGGCGCTGAGGCGGCTG   89,521,035

Hs 154,869,402  AGGGGCTTCATGACCCCACCGCTATACTTGCAGGAGCTCATGGCGATCTCCGTGAAGGGG  154,869,461
Rm 129,130,720  AGGGGCTTCATGACCCCACCGCTATACTTGCAGGAGCTCATGGCGATCTCCGTGAAGGGG  129,130,779
Mm  89,521,034  AGGGGCTTCATGACCCCACCGCTGTATTTGCAGGAGCTCATAGCTATCTCCGTGAAGGGA   89,520,975

Hs 154,869,462  TTGCTGTCCCGCCGGTGCACCAGGGGGCTGGCCTGTCGGTGCCGGCTGCCTCCGCCAGGC  154,869,521
Rm 129,130,780  TTGCTGTCCCGCCGGTGCACCAGGGGGCTGGCCTGCCGGTGCCGGCTGCCTCCGCCAGGC  129,130,839
Mm  89,520,974  TTGCTGTCCCGCCGGTGCACCAGGGGGCTGGCCTGCCGGTGCCGGCTGCCTCCACCGGGC   89,520,915

Hs 154,869,522  CCACTTGTAGCTGTGGAACTTGGAGAGTGGCCAAGCAAGTGGTCATTGAGATTGAGCTGG  154,869,581
Rm 129,130,840  CCACTTGTAGCTGTGGAACTTGGAGAGTGGCCAAGCAAGTGGTCATTGAGATTGAGCTGG  129,130,899
Mm  89,520,914  CCACTTGTGGCTGTGGAACTTGGAGAGTGGCCAAGCAAGTGGTCATTGAGATTTAGCTGG   89,520,855

Hs 154,869,582  CTGCCTTGCCTGGAGGAAGGGTGGAGGATGGCGGTGGAGTTGGACGAAGGGGGGGCCCTG  154,869,641
Rm 129,130,900  CTGCCTTGCCTGGAGGAAGGGTGGAGGATGGCGGTGGAGTTGGACGAAGGGGGGGGCCTG  129,130,959
Mm  89,520,854  CTGCCTTGCCTGGAGGAAGGGTGGAGGATGGCGGTAGAGTTGGCTGAAGTGGGGGCCCTG   89,520,795

Hs 154,869,642  AAAGCGGTGGGAGAGGAGTGCAGCAGGCCAGGGTGGACGGGCTGGCTCTGGAGTTGGGCG  154,869,701
Rm 129,130,960  AAAGCGGTGGGAGAGGAGTGCAGCAGGCCAGGGTGGACGGGCTGGCTCTGGAGTTGGGCG  129,131,019
Mm  89,520,794  AACGCCGTGGGAGAAGAGTGCAACAGACC–AGGATGGAC–AAGCTGGCTCTGGAGTTGGGCA   89,520,735
```

Supplemental Fig. 2. Sequence homology of MR-ex 1 (exon 1/intron 1) across human, macaque and mouse. The start and stop of the differentially methylated region spanning 646 base pairs is shown by green and red arrows, respectively. Conserved CpGs within MR-ex1 are shown in bold, red font. CpGs unique to a species are underlined. Nucleotides that are not conserved across species are shown in italic. '*' and '*' are CpGs that are significantly different in HD/VHD monkeys and CIE-exposed drinking mice, respectively.

Supplemental Figure 3

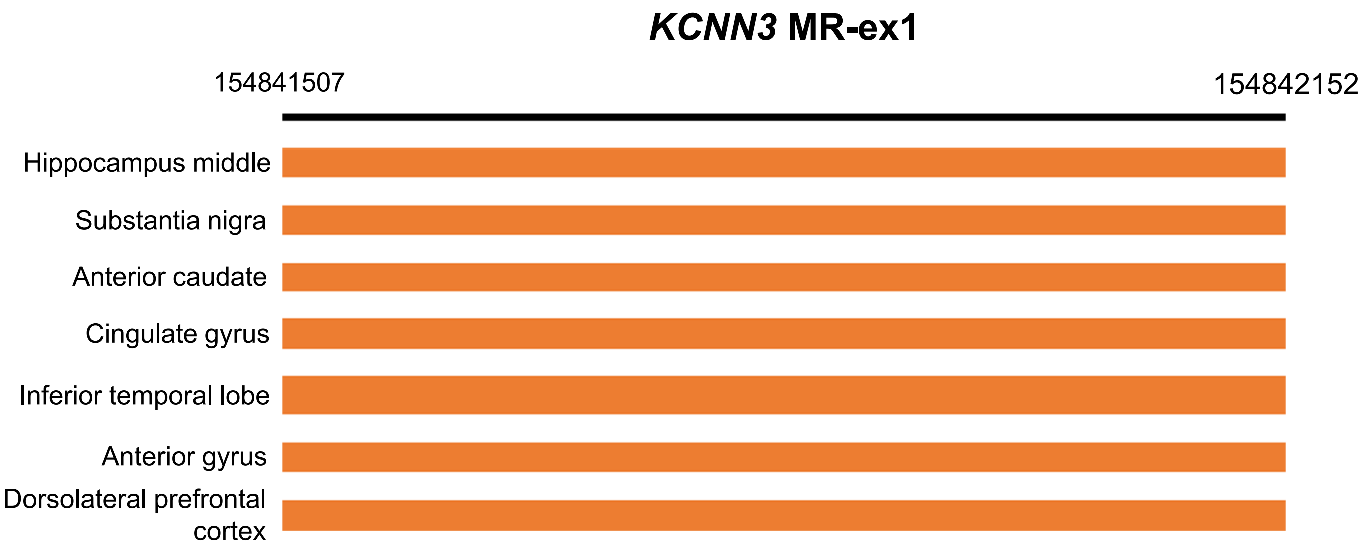

Supplemental Fig. 3. Overlap of the macaque homologous methylation region within MR-ex1 on the human Roadmap 25 chromatin states. The MR-ex1 region on the human genome (hg19) is located at chr1: 154,841,507-154,842,152. According to the Roadmap Epigenomic project, the orange color in the 25 chromatin states represents “promoter upstream transcription start site”. Note that the nucleus accumbens in not included in the Epigenomics Roadmap database.

Supplemental Figure 4

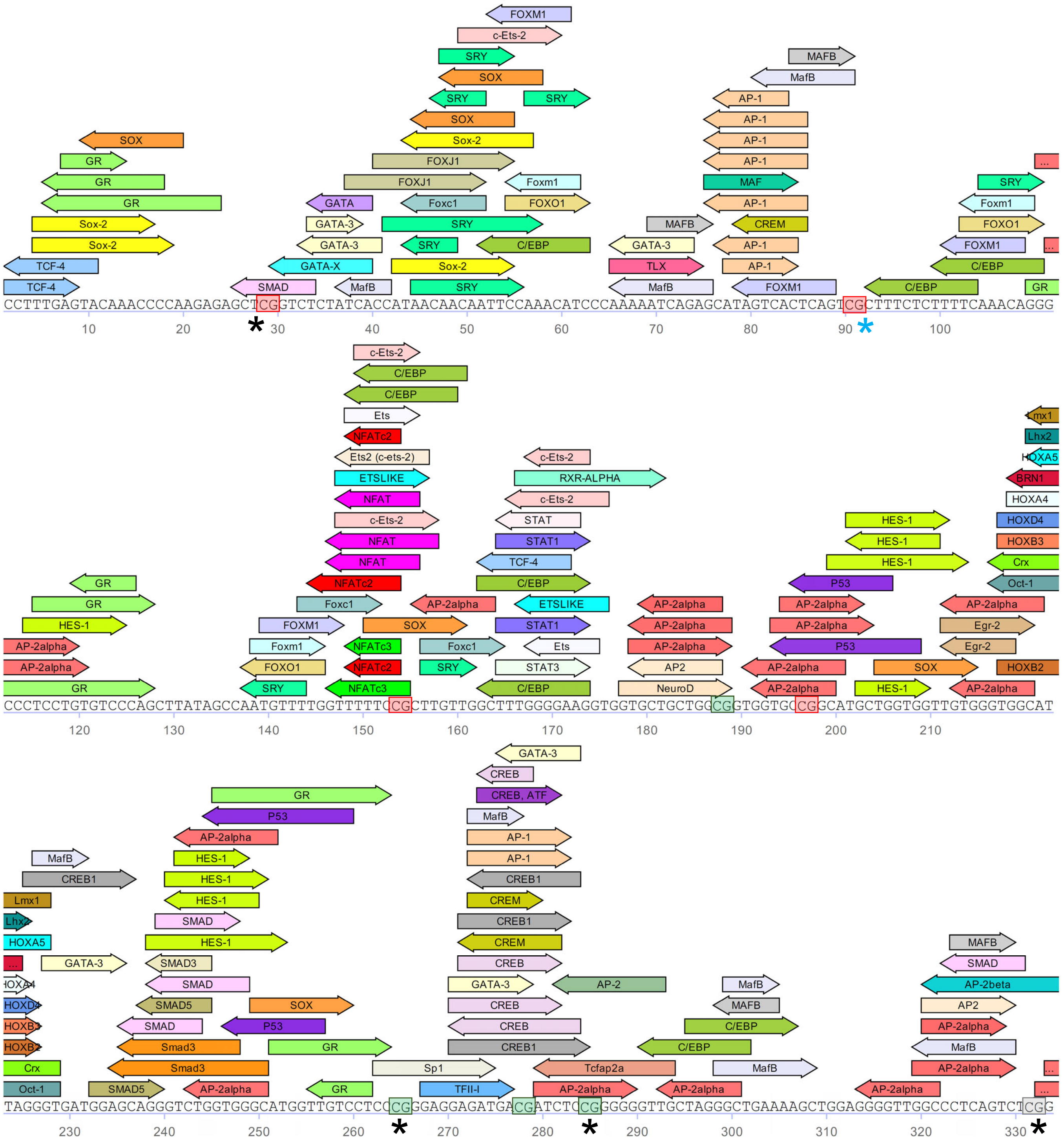

Supplemental Fig. 4. Predicted transcription factors that bind to the human MR-ex1 (TRANSFAC). CpGs enclosed in a red box are conserved in human, monkey and mouse; those CpGs unique to human are enclosed in a grey box, and those conserved in human and monkey are enclosed in a green box. ‘*’ and ‘*’ are CpGs that are significantly different in HD monkeys and CIE-exposed drinking mice, respectively. TRANSFAC conditions were as follows: nerve_system_specific matrices, matrix similarity cut-off: 0.9 and core similarity cut-off: 0.95.

Supplemental Figure 4, cont.

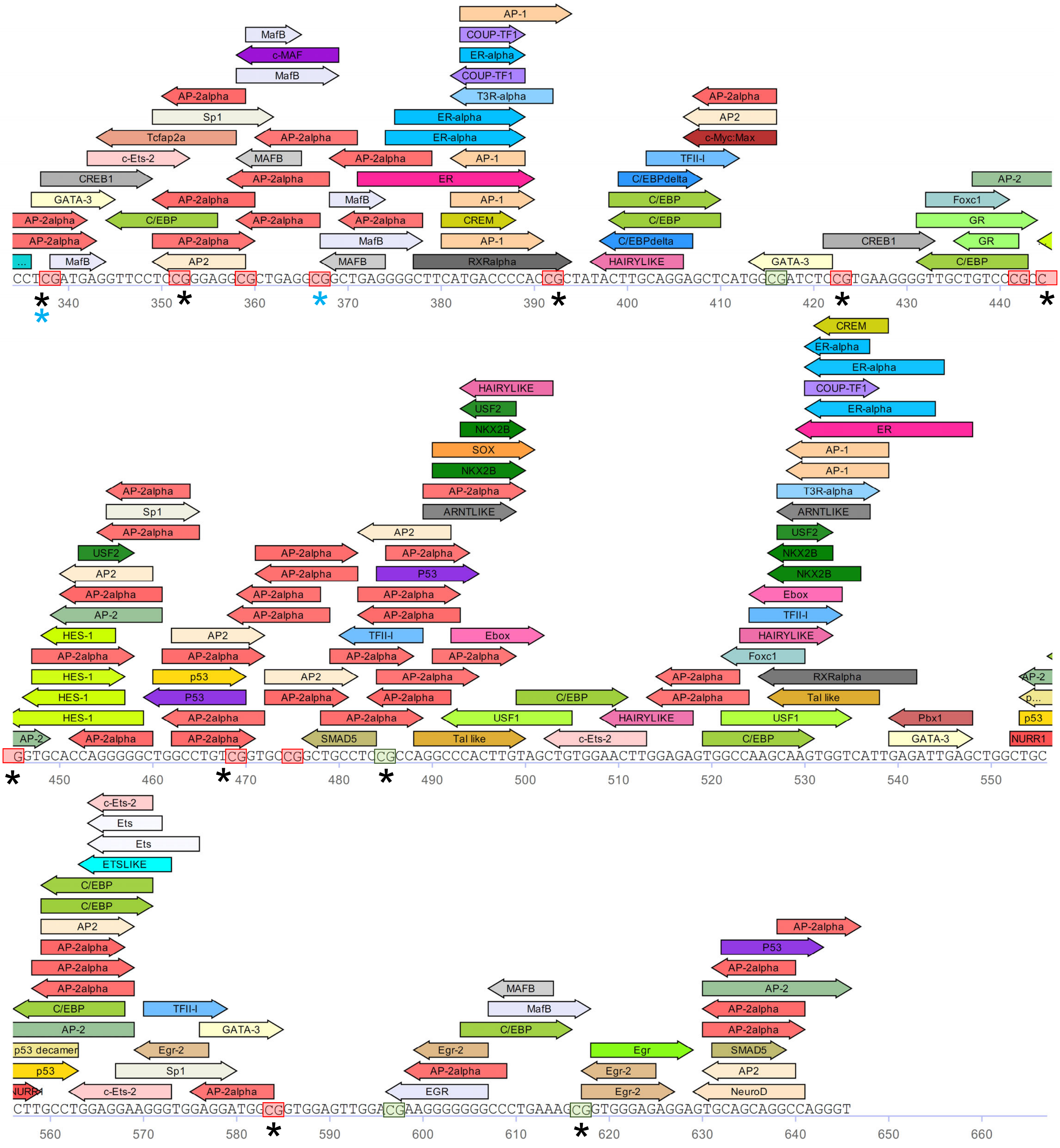

Supplemental Figure 5

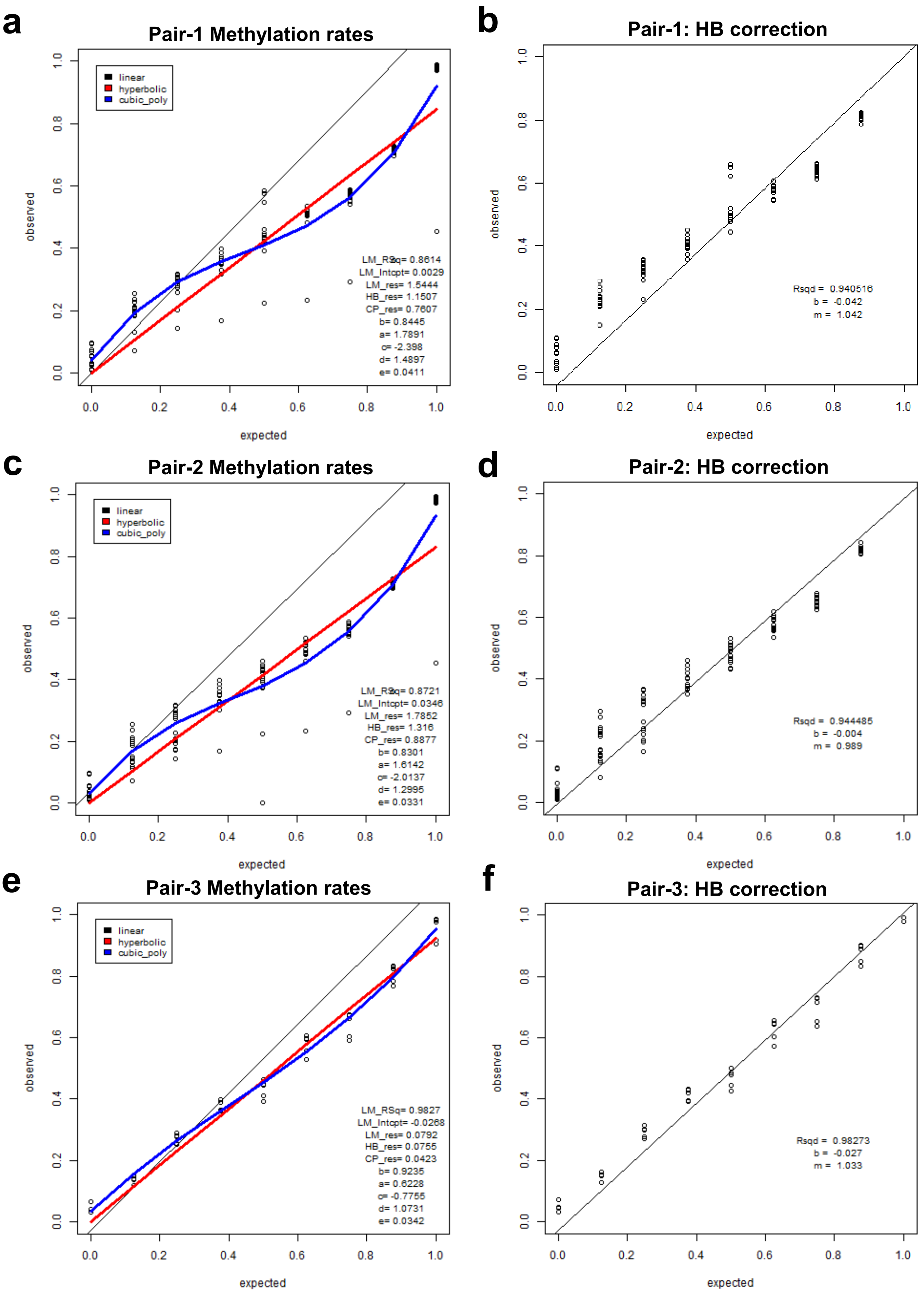

Supplemental Fig. 5. Titration curves to correct for PCR bias during amplification of the methylation region under study. Three different amplicons were used to amplify the complete region 1 (**a**), 2 (**c**) and 3 (**e**). PCR-bias was corrected using the hyperbolic function (HB, **b, d** and **f**).
